# Supplementary material for: The Shigella kinase effector OspG modulates host ubiquitin signaling to escape septin-cage entrapment
Source: Nat Commun. 2024 May 8;15:3890. doi: 10.1038/s41467-024-48205-4 (PMC11078946; doi:10.1038/s41467-024-48205-4)
Supplement: Supplementary file 1 — Supplementary Information [file 41467_2024_48205_MOESM1_ESM.pdf]

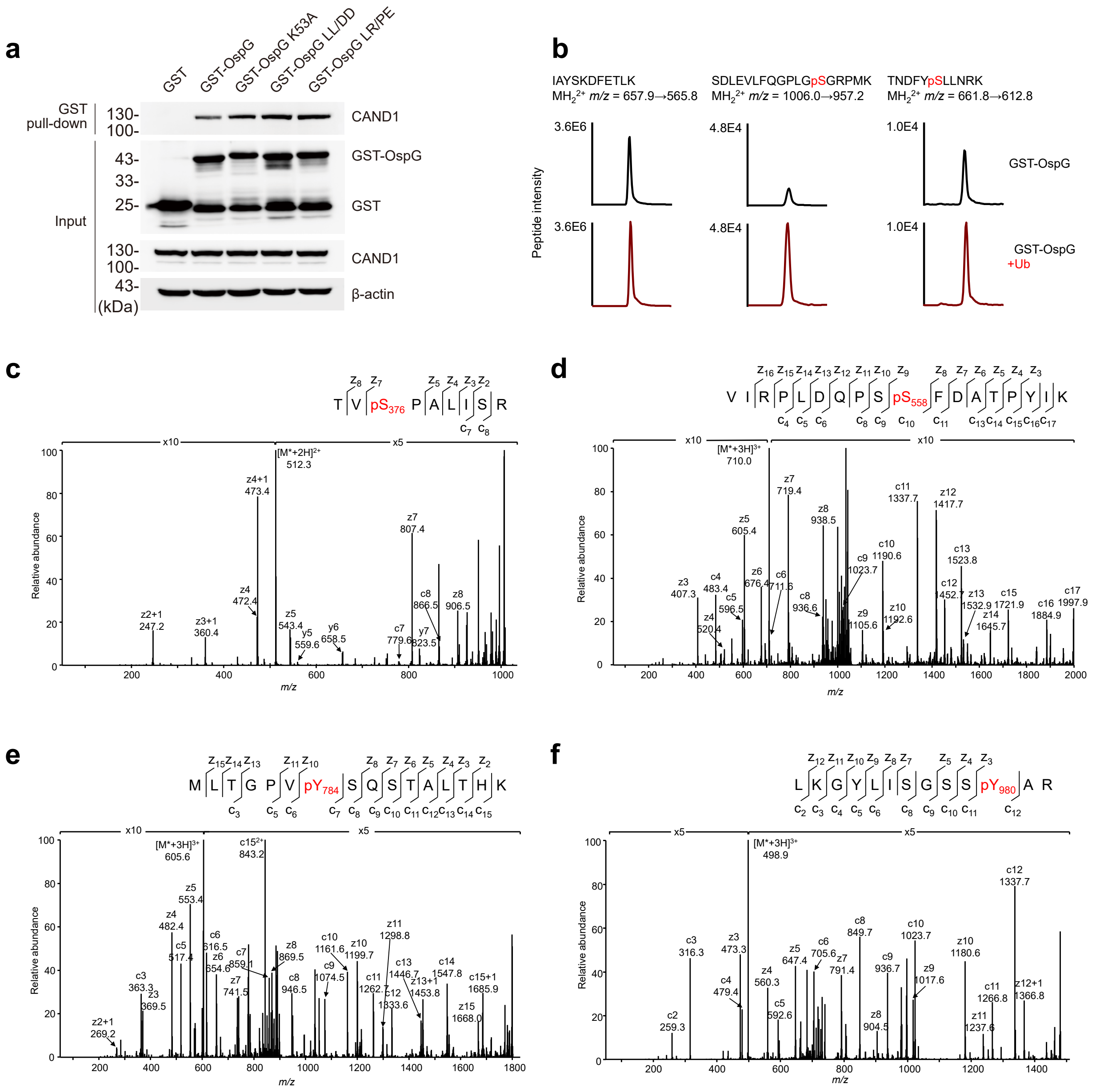

Supplementary Fig. 1 CAND1 is a kinase substrate of OspG. | (**a**) GST pull-down analyses of CAND1 association with OspG or the indicated variants. Glutathione resins coated with GST-OspG or GST alone were incubated with 293T cell lysates. Eluted samples were blotted with anti-CAND1 and anti-GST antibodies. Images are representative of *n* = 2 independent experiments. (**b**) Ub binding stimulates the autophosphorylation of OspG. Purified GST-OspG protein was incubated with ATP with or without the addition of Ub *in vitro*. The samples were subjected to SDS-PAGE separation and subsequent SRM analysis. Extracted ion chromatograms of phosphorylation containing peptides (SDLEVLFQGPLGSGRPMK, TNDFYSLLNRK) and a control peptide (IAYSKDFETLK) of GST-OspG are shown. (**c-f**) Electron transfer dissociation (ETD)-tandem mass spectra of CAND1 peptides bearing phosphorylation at Ser376, Ser558, Tyr784, Tyr980.

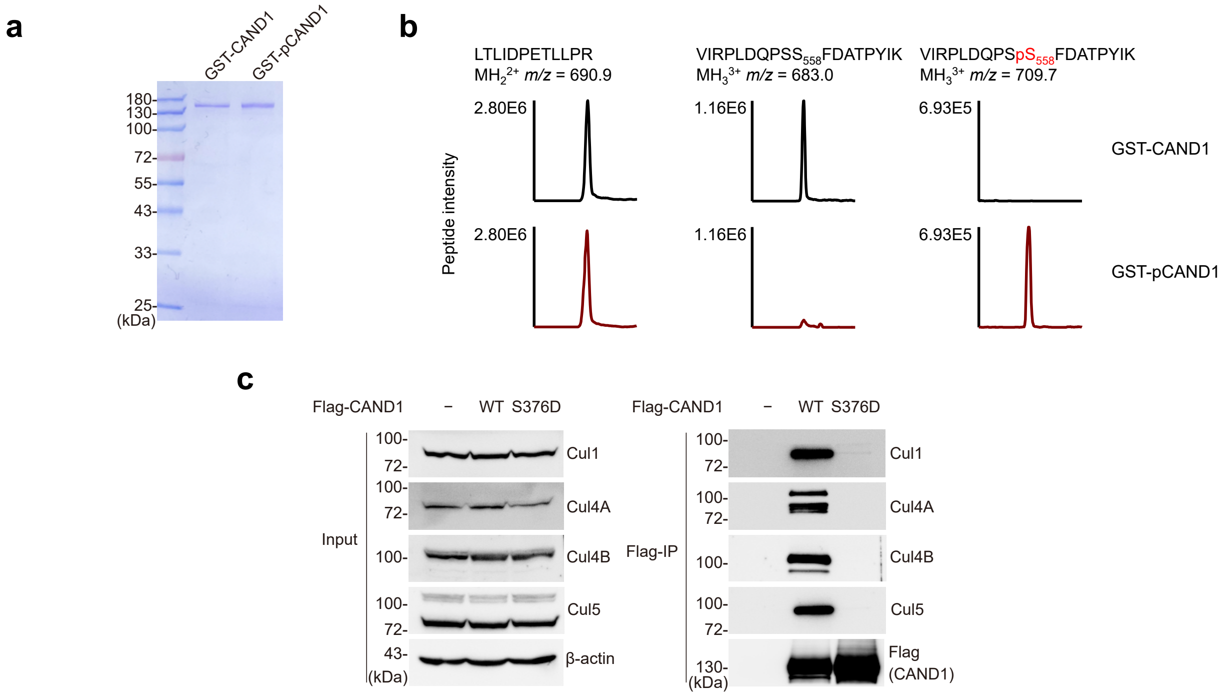

Supplementary Fig. 2 OspG-catalyzed phosphorylation of CAND1 disrupts its interactions with cullins. | (**a**) Coomassie blue staining of purified GST-CAND1 and GST-pCAND1 used in this study. (**b**) Extracted ion chromatograms of a control peptide (LTLIDPETLLPR), the Ser558-phosphorylated peptide (VIRPLDQP-SpS$_{558}$FDATPYIK) as well as its unmodified form (VIRPLDQPSSFDATPYIK) of recombinant GST-CAND1 or GST-pCAND1 purified from *E. coli*. Compared with the control group, the unmodified peptide was barely detected in pCAND1, indicating a high rate of protein modification (close to 100%). (**c**) 293T cells were transfected with Flag-CAND1 or Flag-CAND1 S376D, and immunoprecipitated CAND1 samples were probed with indicated antibodies to assess CAND1-cullin interactions. Images are representative of n = 3 independent experiments.

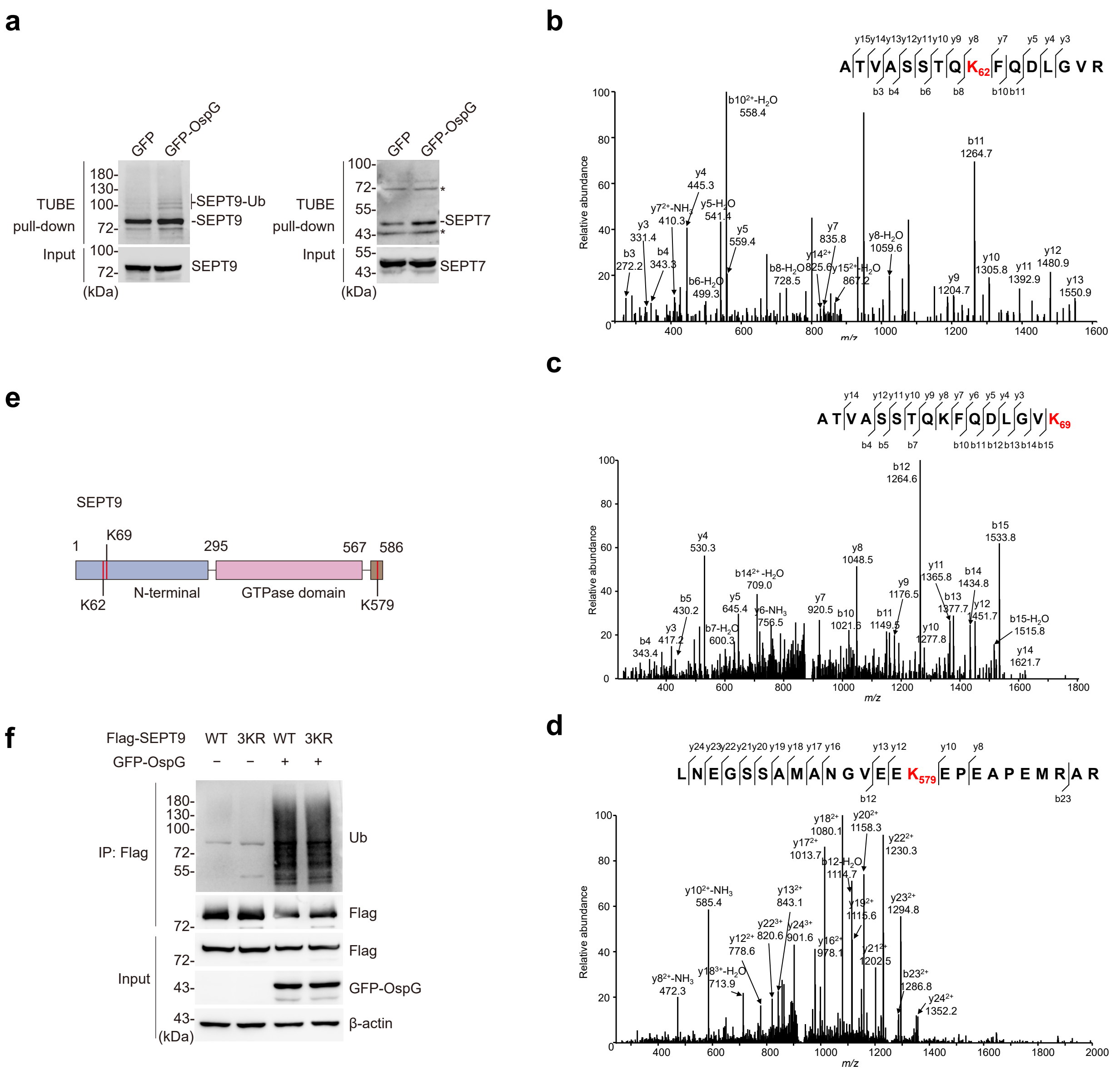

Supplementary Fig. 3 The septin family proteins exhibit elevated ubiquitination levels in an OspG-dependent manner. | (**a**) Immunoblots of TUBE-captured cell extracts showing the signals of SEPT9 or SEPT7 together with their ubiquitinated forms in OspG-expressing or control cells. *, nonspecific bands. Images are representative of $n = 2$ independent experiments. (**b-d**) CID-tandem mass spectra of SEPT9 peptides bearing di-glycine residues at Lys62, Lys69, and Lys579. (**e**) Ubiquitination sites of SEPT9 identified by LC-MS analysis. (**f**) Ubiquitination levels of SEPT9 (WT or 3KR) in 293T cells co-expressing GFP-OspG. Lysates were immunoprecipitated with Flag antibody and immunoblotted as indicated. 3KR denotes a SEPT9 mutant in which all three lysine (K62, K69, K579) residues were replaced with arginine. Images are representative of n = 2 independent experiments.

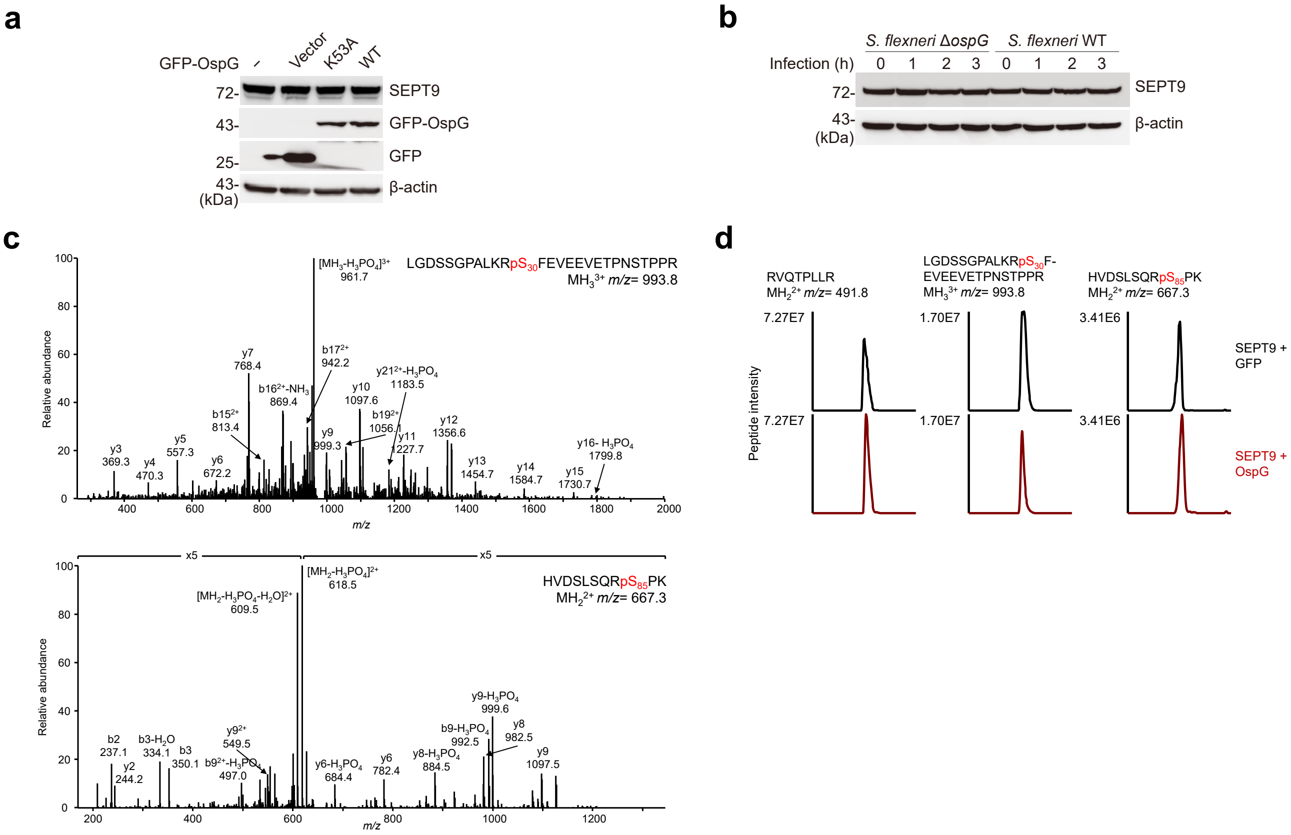

Supplementary Fig. 4 Septin family proteins undergo increased ubiquitination with mixed types of Ub chains. | (**a**) Untransfected 293T cells or those transfected with GFP, GFP-OspG or GFP-OspG K53A for 24 h were lysed and immunoblotted with indicated antibodies. Images are representative of *n* =3 independent experiments. (**b**) Immunoblots of lysate samples from HeLa cells infected with *S. flexneri* strains as indicated. Images are representative of *n* = 3 independent experiments. (**c**) CID-tandem mass spectra of phosphorylated SEPT9 peptides at Ser30, and Ser85. (**d**) Extracted ion chromatograms of phosphorylated peptides at Ser30 (LGDSSGPALKRSFEVEEVETPNSTPPR), Ser85 (HVDSLSQRSPK) and a control peptide (RVQTPLLR) of SEPT9. 293T cells were co-transfected with Flag-SEPT9 and OspG or GFP, and immunoprecipitated SEPT9 samples were resolved by SDS-PAGE before LC-MS analysis.

a

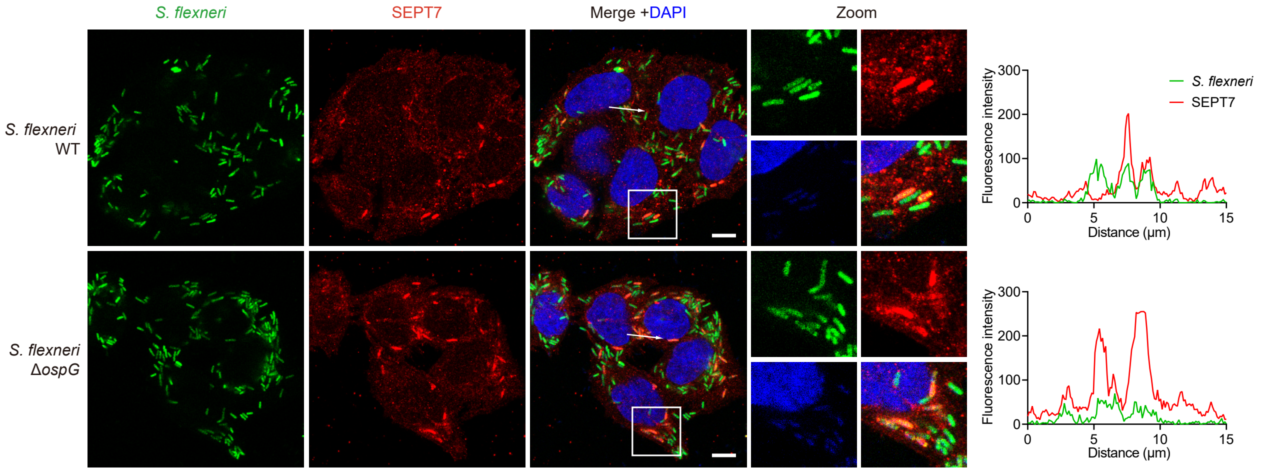

b

Protein	Mass (kDa)	Spectral counts					
		WT rep1	WT rep2	Δ*ospG* rep1	Δ*ospG* rep2	Δ*mxiE* rep1	Δ*mxiE* rep2
LolA	22.5	42	43	36	41	41	41
MxiE	24.5	27	26	31	32	0	0
OspG	22.6	16	10	0	0	10	6

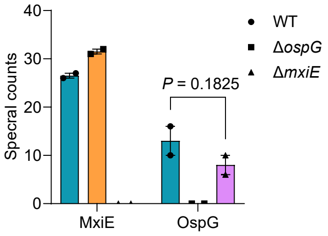

Supplementary Fig. 5 | (**a**) Representative immunofluorescence images of *S. flexneri*-infected cells. HeLa cells were infected with indicated GFP-labelled S. flexneri strains for 2 h, fixed and stained with an anti-SEPT7 antibody. Nuclei were stained with DAPI. Fluorescence intensity was plotted along the arrows. Scale bars, 10 μm. (**b**) Proteomics analysis of *S. flexneri* WT, Δ*ospG* and Δ*mxiE* strains, the outer-membrane lipoprotein carrier protein (LolA) was used as a control. Data are from $n = 2$ independent experiments. Unpaired two-sided Student's *t*-test was performed for statistical analysis.

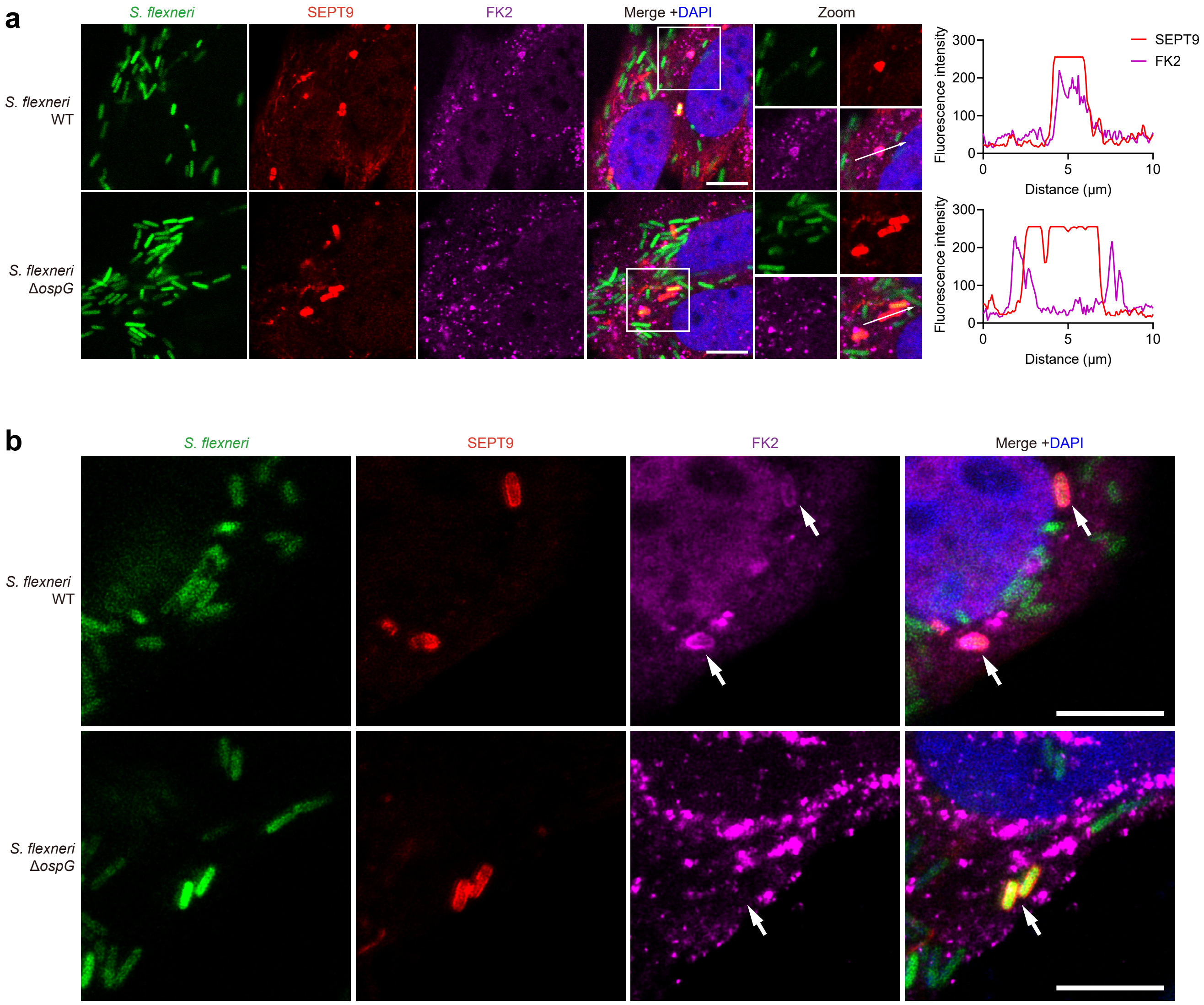

Supplementary Fig. 6 OspG-promoted ubiquitination of septins prevents the assembly of septin-cages around cytosolic *S. flexneri* in infected host cells. | (**a**) Immunofluorescence images of *S. flexneri*-infected cells. HeLa cells were infected with indicated GFP-labelled *S. flexneri* strains for 2 h, fixed and stained with anti-SEPT9 and FK2 antibodies. Nuclei were stained with DAPI. Fluorescence intensity was plotted along the arrows. Scale bars, 10 μm. Images are representative of $n = 2$ independent experiments. (**b**) Immunofluorescence images of *S. flexneri*-infected cells. HeLa cells were infected with indicated GFP-labelled *S. flexneri* strains for 2 h, fixed and stained with anti-SEPT9 and FK2 antibodies. Nuclei were stained with DAPI. For higher magnification, the Lightning 3D deconvolution method was used for image acquisition. Scale bars, 10 μm. Images are representative of $n = 2$ independent experiments.
