# Supplementary material for: The Shigella kinase effector OspG modulates host ubiquitin signaling to escape septin-cage entrapment
Source: Nat Commun. 2024 May 8;15:3890. doi: 10.1038/s41467-024-48205-4 (PMC11078946; doi:10.1038/s41467-024-48205-4)
Supplement: Supplementary file 3 — Description of Additional Supplementary Files [file 41467_2024_48205_MOESM3_ESM.pdf]

Description of Additional Supplementary Files

File Name: Supplementary Data 1
Description: OspG phosphoproteomics

File Name: Supplementary Data 2
Description: GST-CAND1 pull-down

File Name: Supplementary Data 3
Description: Ubiquitome
