# Supplementary material for: The Shigella kinase effector OspG modulates host ubiquitin signaling to escape septin-cage entrapment
Source: Nat Commun. 2024 May 8;15:3890. doi: 10.1038/s41467-024-48205-4 (PMC11078946; doi:10.1038/s41467-024-48205-4)
Supplement: Supplementary file 7 — Reporting Summary [file 41467_2024_48205_MOESM7_ESM.pdf]

nature portfolio

Corresponding author(s): Xiaoyun Liu, Zhao-qing Luo

Last updated by author(s): Mar 6, 2024

Reporting Summary

Nature Portfolio wishes to improve the reproducibility of the work that we publish. This form provides structure for consistency and transparency in reporting. For further information on Nature Portfolio policies, see our Editorial Policies and the Editorial Policy Checklist.

Statistics

For all statistical analyses, confirm that the following items are present in the figure legend, table legend, main text, or Methods section.

n/a	Confirmed	
☐	☒	The exact sample size (*n*) for each experimental group/condition, given as a discrete number and unit of measurement
☐	☒	A statement on whether measurements were taken from distinct samples or whether the same sample was measured repeatedly
☐	☒	The statistical test(s) used AND whether they are one- or two-sided<br>*Only common tests should be described solely by name; describe more complex techniques in the Methods section.*
☒	☐	A description of all covariates tested
☐	☒	A description of any assumptions or corrections, such as tests of normality and adjustment for multiple comparisons
☐	☒	A full description of the statistical parameters including central tendency (e.g. means) or other basic estimates (e.g. regression coefficient) AND variation (e.g. standard deviation) or associated estimates of uncertainty (e.g. confidence intervals)
☐	☒	For null hypothesis testing, the test statistic (e.g. *F*, *t*, *r*) with confidence intervals, effect sizes, degrees of freedom and *P* value noted<br>*Give P values as exact values whenever suitable.*
☒	☐	For Bayesian analysis, information on the choice of priors and Markov chain Monte Carlo settings
☒	☐	For hierarchical and complex designs, identification of the appropriate level for tests and full reporting of outcomes
☒	☐	Estimates of effect sizes (e.g. Cohen's *d*, Pearson's *r*), indicating how they were calculated

Our web collection on statistics for biologists contains articles on many of the points above.

Software and code

Policy information about availability of computer code

Data collection	Leica LAS X software (v3.5.6) was used to acquire confocal images.<br>Proteomics raw data were collected on LTQ Velos Pro or LTQ Orbitrap Velos instrument with Thermo Xcalibur software (v2.2).
Data analysis	Graphing and statistical analysis: Image J software (v1.48),Graphpad Pism (v9.5), R (version 4.1.3) with packages Deseq2 (v1.34.0), pheatmap (v1.0.12),clusterProfiler (v4.2.2) and ggplot2 (v3.4.4), and Adobe Illustrator 2024(v28.0).<br>Mass spectrometry data were processed with Mascot software (v2.3.02, Matrix Science) and MaxQuant (v2.4.10.0).

For manuscripts utilizing custom algorithms or software that are central to the research but not yet described in published literature, software must be made available to editors and reviewers. We strongly encourage code deposition in a community repository (e.g. GitHub). See the Nature Portfolio guidelines for submitting code & software for further information.

Data

Policy information about availability of data

All manuscripts must include a data availability statement. This statement should provide the following information, where applicable:

- Accession codes, unique identifiers, or web links for publicly available datasets
- A description of any restrictions on data availability
- For clinical datasets or third party data, please ensure that the statement adheres to our policy

MS/MS spectra were searched against UniProt human protein database (UP000005640).

The mass spectrometry proteomics data have been deposited to the ProteomeXchange Consortium (https://proteomecentral.proteomexchange.org) via the iProX partner repository with the dataset identifier PXD048447. The link to access the data: https://www.iprox.cn/page/project.html?id=IPX0007864000.
The phosphoproteomics, GST-CAND1 pull-down and ubiquitome search result are provided in the Supplementary Information.
Source data are provided with this paper.

Research involving human participants, their data, or biological material

Policy information about studies with human participants or human data. See also policy information about sex, gender (identity/presentation), and sexual orientation and race, ethnicity and racism.

Reporting on sex and gender	No human research participant in this study.
Reporting on race, ethnicity, or other socially relevant groupings	No human research participant in this study.
Population characteristics	No human research participant in this study.
Recruitment	No human research participant in this study.
Ethics oversight	No human research participant in this study.

Note that full information on the approval of the study protocol must also be provided in the manuscript.

Field-specific reporting

Please select the one below that is the best fit for your research. If you are not sure, read the appropriate sections before making your selection.

☒ Life sciences ☐ Behavioural & social sciences ☐ Ecological, evolutionary & environmental sciences

For a reference copy of the document with all sections, see nature.com/documents/nr-reporting-summary-flat.pdf

Life sciences study design

All studies must disclose on these points even when the disclosure is negative.

Sample size	No statistical methods were used to predict sample size. Our experiments involved transfection and infection in cultured cells, where sample size is not relevant. In proteomics, obtaining sufficient signal intensity is a determinant of how much cell volume to use.<br>To calculate the percentage of septin cage, we referred to the study by Krokowski S et al (PMID: 30543779). Three biological replicates of the experiment were performed and a total of 150 HeLa cells were counted for the amount of Shigella and septin cage.
Data exclusions	No data generated was excluded from initial analysis, and full data is available in public data repositories.
Replication	All attempts at replication were successful. The number of biological replicates contributing to each analysis are indicated in the figure legends.
Randomization	Sample randomization was not relevant to our study. All mass spectrometry data were acquired as biological samples were generated and processed, all samples need to be processed to ensure that the sample name and source are correct and that randomization has no effect on the results of the mass spectrometry data analysis. Randomization is not standard for the other experiments performed, which contained multiple steps requiring distinct and skillful operations, typical in a large number of samples. The need for accuracy, precisions, and data logging in these biochemical and microbiological experiments is not consistent with randomization.
Blinding	Blinding was not required for the proteomic analysis because the result is not predictable until the mass spectrometry data are acquired and being analyzed. For imaging data, no blinding was required because the possibility of biases affecting interpretation of results was managed by means of controls and statistical analysis.

Reporting for specific materials, systems and methods

We require information from authors about some types of materials, experimental systems and methods used in many studies. Here, indicate whether each material, system or method listed is relevant to your study. If you are not sure if a list item applies to your research, read the appropriate section before selecting a response.

Materials & experimental systems

n/a	Involved in the study	
☐	☒	Antibodies
☐	☒	Eukaryotic cell lines
☒	☐	Palaeontology and archaeology
☒	☐	Animals and other organisms
☒	☐	Clinical data
☒	☐	Dual use research of concern
☒	☐	Plants

Methods

n/a	Involved in the study	
☒	☐	ChIP-seq
☒	☐	Flow cytometry
☒	☐	MRI-based neuroimaging

Antibodies

Antibodies used

The following primary antibodies were used in this study: anti-Cul1 (WB 1:200, Santa Cruz, sc-12761), anti-Cul4A (WB 1:5000, abcam, ab92554), anti-Cul4B (WB 1:1000, Abclonal, A12696), anti-Cul5 (WB 1:5000, abcam, ab184177), anti-SEPT7 (IF 1:100, WB 1:2000, Proteintech, 13818-1-AP), anti-SEPT9 (IF 1:300, WB 1:3000, Atlas Antibodies, HPA042564), anti-ubiquitinated proteins, clone FK2 (IF 1:400, Sigma, 04-263), anti-Ubiquitin (WB 1:200, P4D1) (Santa Cruz, sc-8017), anti-CAND1 (WB 1:5000, abcam, ab183748), anti-Flag (WB 1:3000, Zen Bioscience, R24091), anti-HA (WB 1:5000, Invitrogen, 26183), anti-GST (WB 1:2500, Zen Bioscience, 390028), anti-GFP (WB 1:5000, Easybio, BE2001), anti-β-actin (WB 1:2000, Cell Signaling Technology, 4967). The secondary antibodies horseradish peroxidase (HRP)-conjugated anti-mouse IgG (WB 1:5000,115-035-003), HRP-conjugated anti-rabbit IgG (WB 1:5000, 111-035-003) and Cyanine3 (Cy3)-conjugated anti-rabbit IgG (IF 1:500, 111-165-003) were purchased from Jackson ImmunoResearch Laboratories. Alexa Fluor 647-labeled anti-mouse IgG (IF 1:500, A0473) were purchased from Beyotime biotechnology.

Validation

1. anti-Cul1 (Santa Cruz, sc-12761): Application statement in manufacturer's website as follows: https://datasheets.scbt.com/sc-12761.pdf

2. anti-Cul4A (abcam, ab92554) : Application statement in manufacturer's website as follows: https://www.abcam.com/products/primary-antibodies/cullin-4acul-4a-antibody-epr3198-ab92554.html

3. anti-Cul4B (Abclonal, A12696) : Application statement in manufacturer's website as follows: https://abclonal.com.cn/Datasheet/Antibodies/A12696.pdf?v=1696639345

4. anti-Cul5 (abcam, ab184177) : Application statement in manufacturer's website as follows: https://www.abcam.cn/products/primary-antibodies/cullin-5cul-5-antibody-epr14725-ab184177.html

5. anti-SEPT7 (Proteintech, 13818-1-AP) : Application statement in manufacturer's website as follows: https://www.ptgcn.com/Products/CISD2-Antibody-13318-1-AP.htm

6. anti-SEPT9 (Atlas Antibodies, HPA042564) : Application statement in manufacturer's website as follows: https://www.sigmaaldrich.cn/CN/zh/product/sigma/hpa042564

7. anti-ubiquitinated proteins, clone FK2 (Sigma, 04-263) : Application statement in manufacturer's website as follows: https://www.sigmaaldrich.cn/CN/zh/product/mm/04263

8. anti-Ubiquitin (P4D1) (Santa Cruz, sc-8017) : Application statement in manufacturer's website as follows: https://datasheets.scbt.com/sc-8017.pdf

9. anti-CAND1 (abcam, ab183748) : Application statement in manufacturer's website as follows: https://www.abcam.cn/products/primary-antibodies/cand1-antibody-epr14241-n-terminal-ab183748.html

10. anti-Flag (Zen Bioscience, R24091) : Application statement in manufacturer's website as follows: http://www.zen-bio.cn/prod_view.aspx?IsActiveTarget=True&TypeId=189&Id=561073&FId=t3:189:3

11. anti-HA (Invitrogen, 26183) : Application statement in manufacturer's website as follows: https://www.thermofisher.cn/cn/zh/antibody/product/HA-Tag-Antibody-clone-2-2-2-14-Monoclonal/26183

12. anti-GST (Zen Bioscience, 390028) : Application statement in manufacturer's website as follows: http://www.zen-bio.cn/prod_view.aspx?IsActiveTarget=True&TypeId=189&Id=561013&FId=t3:189:3

13. anti-GFP (Easybio, BE2001) : Application statement in manufacturer's website as follows: http://bioeasytech.com/product/2407.html

14. anti-β-actin (Cell Signaling Technology, 4967) : Application statement in manufacturer's website as follows: https://www.cellsignal.com/products/primary-antibodies/b-actin-antibody/4967

15. HRP-conjugated anti-mouse IgG (115-035-003) : Application statement in manufacturer's website as follows: https://www.jacksonimmuno.com/catalog/products/115-035-003

16. HRP-conjugated anti-rabbit IgG (111-035-003) : Application statement in manufacturer's website as follows: https://www.jacksonimmuno.com/catalog/products/111-035-003

17. Cyanine3 (Cy3)-conjugated anti-rabbit IgG (111-165-003) : Application statement in manufacturer's website as follows: https://www.jacksonimmuno.com/catalog/products/111-165-003

18. Alexa Fluor 647-labeled anti-mouse IgG (A0473) : Application statement in manufacturer's website as follows: https://www.beyotime.com/product/A0473.htm

Eukaryotic cell lines

Policy information about cell lines and Sex and Gender in Research

Cell line source(s)	293T (Cat No. CRL-3216) and HeLa cells (Cat No. CRM-CCL-2) were obtained from American Type Culture Collection (ATCC).
Authentication	Cells were purchased and received directly from their respective manufacturer. Authentication was performed by the manufacturer, no additional in-house authentication was performed.
Mycoplasma contamination	Negative mycoplasma tests for all cell lines were certified by the manufacturers, no additional in-house test for mycoplasma contamination but no indication of contamination was observed.
Commonly misidentified lines (See ICLAC register)	No commonly misidentified lines were used in this study.

Plants

Seed stocks	No plants were used in this study.
Novel plant genotypes	No plants were used in this study.
Authentication	No plants were used in this study.
